# Effects of an Equine-Assisted Riding Program on Motor Performance, Movement Quality, and Well-Being Among Young Inmates

**DOI:** 10.3390/healthcare14101418

**Published:** 2026-05-21

**Authors:** Milan Dransmann, Martin Koddebusch, Pamela Wicker, Daniela Gröben, Bernd Gröben

**Affiliations:** 1 Department of Sports Science, Bielefeld University, Universitätsstrasse 25, 33615 Bielefeld, Germany; martin.koddebusch@uni-bielefeld.de (M.K.); pamela.wicker@uni-bielefeld.de (P.W.); bernd.groeben@uni-bielefeld.de (B.G.); 2 Equi-Didaktik, Denkmalstrasse 128, 32760 Detmold, Germany; mail@equi-didaktik.de

**Keywords:** prison, youth detention, horse-riding, rehabilitation, sports, welfare, autotelic, telic, intervention, motor learning

## Abstract

Background: Equine-assisted programs have been shown to promote psychosocial outcomes, but quantitative evidence of motor benefits in correctional settings is scarce. Aim: The present study examined the effects of a one-week equine-assisted riding program on riding performance, movement quality, and well-being among young inmates in an open German prison. Methods: Ten male participants (24.5 ± 0.71 years) completed a five-day program combining practical riding exercises, cooperative activities, and guided reflection. Riding performance was assessed using standardized expert video ratings based on the German performance testing guidelines on a 10-point scale, movement quality using a semantic differential with bipolar adjective pairs assessing telic and autotelic dimensions on a six-point scale, and well-being using the WHO-5 Well-Being Index. A single-group pre–post repeated-measures design without a control group was applied. Results: Significant improvements were found in riding performance for both walk and trot, with large effect sizes (*n* = 10). Participants also reported a significant enhancement in the autotelic, experience-oriented dimension of movement quality, whereas no significant change occurred in the telic, goal-oriented dimension. Well-being increased significantly from pre- to post-test. Conclusions: Even a short, experience-focused equine-assisted program can produce meaningful improvements in motor performance, positive movement experience, and well-being in a correctional context. Equine-assisted programs may therefore represent a promising complementary approach to rehabilitation by integrating physical, emotional, and social learning processes.

## 1. Introduction

Correctional systems serve a dual purpose by both protecting the public through restricting inmates’ liberty and promoting their rehabilitation into society. However, the punitive nature of imprisonment often contradicts rehabilitative objectives, as “the deprivation of liberty leads to the development of pains of imprisonment linked to the loss of autonomy and the reduction of inmates to a childlike state of dependency” [1] (p. 63). This loss of autonomy may weaken self-directed behavior and bodily agency, both of which are important for regular movement practice, active participation, and the development of motor competence [2]. These structural factors can result in prison sickness, characterized by demotivation, lethargy, and an inactive lifestyle [3]. The consequences are particularly evident in reduced physical activity and motor competence, which can negatively affect inmates’ physical health and perceived self-efficacy [4].

From a rehabilitative perspective, the promotion of physical and motor skills is not only a matter of health, but also of empowerment [5]. Motor learning processes can foster self-confidence, self-awareness, and goal orientation, factors that are vital for rehabilitation [6]. Motor learning is not driven by repetition alone, but also by attentional focus, emotional activation, and the meaningful appraisal of movement experiences [7]. Moderate emotional arousal can enhance bodily awareness and task engagement, thereby facilitating the acquisition and consolidation of new motor patterns [8]. In this sense, learning situations that combine physical action with emotionally salient feedback may be particularly effective for individuals with limited prior success experiences in movement-related contexts [7]. This may be especially relevant in correctional settings, where reduced autonomy, low self-efficacy, and emotional dysregulation can interfere with both motivation and skill development [3].

Participation in structured physical activity has been shown to mitigate the pains of imprisonment and enhance overall well-being [9,10,11]. Conventional prison sports primarily focus on physical fitness [12], discipline, and competition, offering limited opportunities for reflection, emotional engagement, or relational learning [13]. To address this gap, programs that include structured human–animal interactions have been introduced in correctional settings, as such interactions can foster empathy, responsibility, and calmness alongside physical activation. Systematic evidence indicates that such interventions can improve emotional regulation and prosocial behavior among inmates [14].

Among different species used in such interventions, horses play a distinctive role because their size, sensitivity, and responsiveness require participants to regulate their emotions and communicate through body language. While most studies have examined the psychosocial or behavioral outcomes of these interventions, one study explored the mechanism of action underlying equine-assisted programs [15]. It was found that successful communication with the horse elicited emotional arousal followed by positive behavioral adaptation, highlighting the potential role of embodied emotional learning in explaining the effects of equine-assisted activities. More specifically, emotional arousal may heighten attentional focus and bodily awareness, thereby increasing sensitivity to one’s own posture, tension, and movement timing. In interaction with a responsive animal such as a horse, these internal adjustments may be followed by immediate external feedback, which can support the modification and consolidation of more functional motor patterns. However, this research did not involve incarcerated participants or assess motor outcomes.

While previous research focused on mechanisms in non-custodial settings, subsequent studies have explored how equine-assisted programs function within correctional environments. One study implemented an equine-assisted learning intervention with young male offenders in a British prison [16]. Participants learned natural horsemanship skills and were observed to develop greater calmness, emotional control, and confidence, which appeared to transfer to daily life on the prison wings. These qualitative findings, derived from observations and interview self-reports, highlight the potential of equine-assisted learning to foster self-management and social competence. Another study described an equine-assisted intervention developed at a women’s correctional facility in Germany [17]. The program targeted young female inmates within a social–therapeutic unit and aimed to promote resocialization through practical work with horses. The intervention emphasized empathy, responsibility, and self-reflection, combining groundwork and riding tasks under therapeutic supervision. Qualitative evaluations and a small ethnographic pilot project indicated improvements in self-perception, social interaction, and trust-building, as well as enhanced cooperation between inmates and staff. A mixed-methods study conducted in the United States examined a prison–equine program called Blue Hills, focusing on incarcerated men, program staff, and community members [18]. The findings showed that participation in equine programs can reduce stigma, strengthen social relationships, and support rehabilitation by fostering responsibility, empathy, and emotional regulation across different stakeholder groups.

Collectively, these studies demonstrate promising psychosocial benefits of equine-assisted programs in correctional contexts, including improved self-control, empathy, and social functioning. However, as highlighted in a review [19], empirical research on equine-assisted prison programs remains limited, with a particular lack of quantitative evaluations of physical or motor outcomes. Specifically, previous studies have not provided objective pre–post assessments of riding-related motor performance, movement quality, or gait-specific riding skills in correctional settings.

Young inmates are of particular interest in this regard. In the present study, this term refers to inmates aged 26 years or younger. As a group, they exhibit higher reoffending rates and greater difficulties in emotional regulation, impulsivity, and social integration than older prisoners [16]. At the same time, they are often first-time offenders with comparatively high prospects for successful rehabilitation, which makes them a particularly relevant target group for rehabilitative interventions in correctional settings [20]. This perspective is consistent with a holistic riding approach, which understands riding not merely as a technical skill, but as an embodied process involving physical coordination, emotional regulation, and relational attunement to the horse [21].

To address these gaps, the present study investigates whether participation in a one-week, experience-focused equine-assisted riding program can improve riding performance and movement quality among young inmates of a German prison. The program design was conceptually rooted in this holistic riding approach [21]. Accordingly, movement quality, motor learning, and well-being were understood as interrelated aspects of the same embodied learning process. By integrating physical, technical, and psychosocial components of equine activity, the study seeks to advance the understanding of how equine-assisted programs can foster rehabilitation through the unity of bodily experience, emotional regulation, and skill acquisition in correctional settings. We hypothesized that participation in the program would be associated with improvements in riding performance, movement quality, and well-being from pre-test to post-test.

## 2. Materials and Methods

### 2.1. Participants

The study was conducted in cooperation with an open prison in Germany as part of a project promoting physical activity among inmates. Participants were recruited through information sessions held by the university.

Participation was voluntary, and all “Jungtäter”, defined by the North Rhine-Westphalian concept as inmates aged 26 years or younger, were invited to take part [22] (p. 105). This age range broadly corresponds to the definition of youth in the Shell Youth Study, which defines young people up to the age of 25 years [23]. Because only men are incarcerated in this prison, all participants in the study were male. All participants were serving their sentences in an open prison, allowing them to leave the prison for work. Depending on individual behavior and security level, temporary leaves of several hours or days were also possible. The prison has a total capacity of 50 inmates, of whom 19 places were designated for “Jungtäter”. From this eligible subgroup, ten individuals volunteered to participate in the riding program (24.5 ± 0.71 years). None of the participants reported any prior riding experience, ensuring a comparable baseline level of motor familiarity with equine interaction across the group. In addition, basic anthropometric characteristics (height, body weight, fat mass, and fat-free mass) were assessed at pre- and post-test to further characterize the sample. These variables were included as descriptive background measures but were not part of the main analyses, as they were not central to the study aims, and the sample size was too small to permit a meaningful examination of their potential role as covariates.

Before the start of the program, all participants were informed about the study design and content, data-handling procedures, and their right to withdraw before providing written informed consent. Neither physical examination nor physician clearance was conducted. All procedures complied with the Declaration of Helsinki, and the study was approved by the Ethics Committee of Bielefeld University (approval number 2022-193).

### 2.2. Design and Procedure

**Figure 1:**
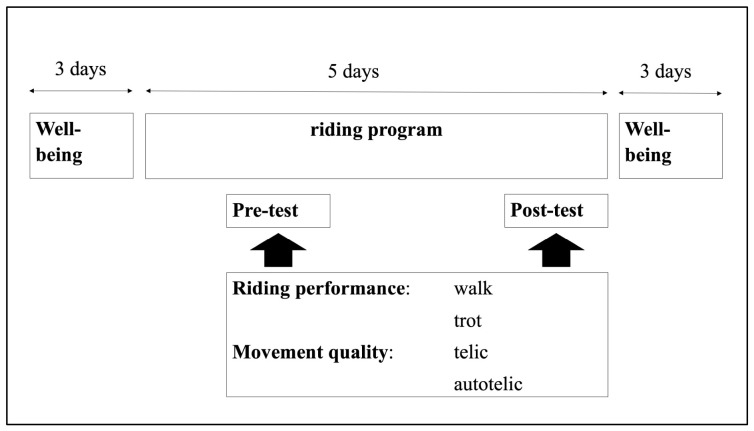
Study design and outcome variables.

The investigation followed a pre–post design to examine changes in riding performance, movement quality, and well-being resulting from a one-week equine-assisted program. This design was chosen because it allows the detection of short-term intra-individual changes while controlling for inter-individual variability [24]. Figure 1 provides an overview of the study design and the assessed outcome variables.

After a familiarization day without riding on a live horse, the pre-test was conducted on the second day, the first day of riding, and the post-test took place on the fifth and final day. This procedure was chosen to ensure that participants had sufficient initial contact with the horses and the riding setting to reduce stress and insecurity, while avoiding prior riding experience that could have influenced the baseline assessment. Conducting both assessments at the same time of day minimized circadian effects on physical and psychological outcomes [25]. Movement quality was measured directly after the first and last riding sessions to capture participants’ immediate, embodied perceptions of their riding experience. This timing was chosen because conscious awareness and subjective appraisal of one’s own motor performance can enhance motor-skill learning and efficient movement organization [26]. Well-being was assessed in the prison three days before the start and three days after the final day of the program to reduce situational bias and allow for short-term consolidation of potential effects [27]. The study deliberately relied on field-based assessments conducted at the cooperating riding facility to ensure ecological validity and to capture participants’ authentic behavior within the correctional environment. Laboratory testing procedures (e.g., biomechanical or physiological measures) were deemed unsuitable due to the logistical and ethical constraints of conducting research in a prison setting. All measurements were supervised by the scientific project coordinator and two trained assistants who followed a standardized protocol to ensure objectivity and consistency across all participants [28].

### 2.3. Program

The equine-assisted program was designed following a holistic approach [21]. This concept does not regard the horse as a means of transportation but as a social and emotional partner, emphasizing responsibility, empathy, and closeness in human–animal interaction. The program lasted one week (five consecutive days) and was conducted at a cooperating riding facility. The program was supervised by three certified riding instructors, supported by two correctional officers and three university staff members. Each day followed a standardized structure to ensure comparable conditions for all participants.

On the first day, participants were introduced to the horses on the pasture. They observed the animals, solved small cooperative tasks, and learned how to approach a horse from the ground. This was followed by grooming and basic leading exercises in the riding arena to establish initial trust and familiarity between participants and horses. To prepare for the first riding session, participants then practiced mounting, pelvic coordination, posting trot, and dismounting on a stationary wooden horse. No riding on a live horse took place on the familiarization day. This introductory session was deliberately limited to observation, handling, and exercises on a stationary wooden horse to build confidence in their own abilities, ensure safety for both participants and horses, prevent unnecessary physical strain on the horses, and prepare participants gradually for the first actual riding session.

From the second day onward, all program days followed a similar structure combining riding sessions, farm-related tasks, and guided reflection. To manage logistics and avoid overburdening the animals, participants were randomly assigned to two groups of five. Group A rode in the morning while Group B performed farm-related tasks such as animal care, cleaning, and small farm construction work (e.g., planting trees or building nesting boxes for wild bees). Because all participants alternated systematically between riding-related and farm-related components each day, exposure to these activities was balanced across the group. After a shared lunch and joint stable chores (e.g., mucking out), the groups switched roles. Each session combined practical riding exercises—performed either on a stationary wooden horse, on a live horse ridden on the lunge line and guided by an instructor from the ground, or on a led horse by another participant—focusing on coordination, rhythm, and posture, with cooperative elements encouraging mutual support and problem-solving. Throughout the week, participants also deepened their understanding of equine behavior, including the horse’s needs as a herd and flight animal and its ways of expressing emotions [29]. This ongoing reflection on the horse’s nature was considered essential for interpreting its reactions and fostering a sense of partnership in riding [30].

At the end of each day, participants took part in a guided reflection session, led by one of the coaches, to discuss their experiences, challenges, and learning outcomes. A final group reflection was conducted at the end of the week to evaluate the overall experience and perceived personal development. Daily and final reflection sessions were integrated into the program to promote emotional processing and learning transfer, as reflective practice is a central element of experiential and equine-assisted learning approaches [31]. Moreover, it is considered essential for rehabilitative purposes in general sport programs [4].

The week concluded with a final riding tournament that served as the post-test assessment, providing an opportunity for participants to demonstrate their progress and apply the skills acquired during the program, followed by a closing barbecue to celebrate the completion of the five days in an informal setting.

### 2.4. Riding Performance

Riding performance was recorded on video during the pre- and post-test. At both testing points, one participant rode while another participant led the horse at the walk and trot to ensure safety and controlled movement. To ensure animal welfare, two horses were used during the assessments rather than one single horse for all participants. The horses were used in alternating order, with rest periods between test rides to prevent fatigue and excessive physical strain. All participants followed the same standardized procedure for mounting and riding, and all test rides were conducted along the same predefined line in the arena. Each participant rode the same horse in both the pre- and post-test to minimize horse-related variability across measurement points. All assessments took place on the same outdoor riding arena with a sand surface under standardized conditions regarding time of day, lighting, and arena setup to ensure comparability across testing points. The recordings were evaluated by two independent experts using a standardized rating scale based on the German Leistungs-Prüfungs-Ordnung (performance testing guidelines) [32]. The recordings were presented in randomized order, and the evaluators were blinded to the measurement time point (pre-test vs. post-test). Riding performance was assessed separately for the two gaits, walk and trot. The walk score was based on five items, whereas the trot score was based on four items. In the walk, the assessment focused on the rider’s alignment with the line of balance, hip coordination with the horse’s motion, trunk stability and position, thigh contact and leg position, as well as hand position. In the trot, the evaluation considered thigh and knee action during the rising phase, upper-body control during the landing phase, rhythm throughout the movement cycle, and the position of the lower leg and foot. For each gait, the mean value across the respective items was calculated to obtain a gait-specific performance score. To generate an overall indicator of riding performance, the two gait-specific means were averaged, ensuring equal weighting of both gaits regardless of the number of underlying items. This approach was chosen to represent walk and trot as equally relevant components of overall riding performance. All criteria were rated on a 10-point scale (0 = not executed, 10 = executed excellently). All ratings were completed independently by the two evaluators, and inter-rater reliability was assessed using an intraclass correlation coefficient (ICC; two-way mixed-effects model, absolute agreement, average measures). The analysis was based on 20 paired ratings derived from ten participants assessed at pre- and post-test. The ICC indicated excellent agreement [33] between the two raters, ICC = 0.907, 95% CI [0.767, 0.963].

### 2.5. Movement Quality and Well-Being

Movement quality and well-being were assessed using two standardized self-report instruments. Movement quality was measured using a semantic differential designed to assess both the telic (goal-oriented) and the autotelic (experience-oriented) aspects of movement. At both measurement points, participants were asked to complete the instrument immediately after the riding session in response to the prompt “My riding today was …”.

The instrument included two subscales with five bipolar adjective pairs each, representing evaluative and affective qualities of movement execution. Participants rated each pair on a six-point scale, with higher scores indicating a more positive perception of their movement performance. This semantic differential is based on a theoretical model that conceptualizes movement quality as a two-dimensional construct comprising telic and autotelic aspects. Previous research has empirically supported this model and demonstrated that such a two-factor structure provides a valid and reliable assessment of movement quality across different types of motor tasks [34].

The wording of all items and the internal consistency coefficients for both subscales are presented in Table 1. Following established procedures, mean values were calculated separately for the telic and autotelic subscales to obtain dimension-specific indices. Internal consistency was acceptable for the telic subscale (Cronbach’s α = 0.765) and questionable for the autotelic subscale (Cronbach’s α = 0.610) [35]. The item unstable–balanced was theoretically assigned to the autotelic dimension but showed a low corrected item-total correlation (r = 0.20) and slightly improved internal consistency when excluded (α = 0.610 vs. 0.588). Therefore, it was omitted from the reliability and index calculations.

Well-being was measured with the WHO-5 Well-Being Index [36], which includes five positively phrased items rated on a six-point scale from 0 to 5; higher total scores indicate better well-being. In line with the authors’ recommendations, a total score was calculated by summing the five items, resulting in a possible range from 0 (very low well-being) to 25 (maximum well-being). The internal consistency of the WHO-5 in the present sample was high (Cronbach’s α = 0.87), consistent with previous findings among inmate populations [37]. Well-being was assessed in the prison three days before the start and three days after the final day of the program to reduce acute situational influences of the riding sessions and to capture a more generalized subjective evaluation of well-being in the participants’ usual correctional environment.

### 2.6. Statistical Analyses

Statistical analyses were conducted using IBM SPSS Statistics 30 (IBM Corp., Armonk, NY, USA). Descriptive statistics (means, standard deviations) and exploratory data analyses were first performed to examine variable distributions, identify outliers, and detect potential ceiling or floor effects [24]. The dependent variables included riding performance, telic and autotelic movement quality, and well-being. For all measures, mean or total scores were calculated as described above; higher values indicated more positive outcomes.

Normality was examined using the Shapiro–Wilk test. Although most variables did not show significant deviations from normality (*p* > 0.05), post-test well-being was not normally distributed (*p* = 0.008). Inspection of the distribution indicated a left-skewed pattern, with most participants reporting high well-being after the program and one participant remaining at a very low level. Given the small sample size and to ensure a conservative and consistent analytical approach across outcomes, pre–post comparisons were conducted using Wilcoxon signed-rank tests. Effect sizes were calculated as *r* = *z*/$\sqrt{N}$, following Rosenthal [38], and are reported alongside the test results. In line with Cohen [39], effect sizes of *r* = 0.10, *r* = 0.30, and *r* = 0.50 were interpreted as small, medium, and large, respectively. The significance level was set at α = 0.05.

Given the small sample size (*n* = 10), statistical power was limited; therefore, both significance levels (α = 0.05) and effect sizes were reported. Although this sample size limits statistical power, such constraints are not unusual in applied correctional research, where access to participants is inherently restricted [40]. According to recommendations for small sample sizes [41], the study emphasized reliable measurement procedures and transparent reporting to ensure robust findings despite limited sample sizes.

An a priori power analysis was conducted using G*Power 3.1 for the Wilcoxon signed-rank test (matched pairs). Because no prior studies reported directly comparable effect sizes for this specific design and population, a medium effect size (*d_z_* = 0.50) according to Cohen [39] was assumed. Using a two-tailed test, an alpha level of 0.05, and a power of 0.80, the analysis indicated a required sample size of 35 participants. Such a sample size was not feasible in the present study due to the limited accessibility of the correctional setting and the organizational constraints of the program. Although the prison has a total capacity of 50 inmates, only 19 places are designated for “Jungtäter”. Thus, even if all eligible inmates had volunteered to participate, the required sample size could not have been reached.

Although a multivariate approach would have been valuable to account for the interrelated nature of the outcome variables, it was not appropriate for the present study because the sample size was too small to support a reliable multivariate analysis [42]. While follow-up assessments could have offered valuable insights into the persistence of these effects, the study design had to account for the logistical and ethical constraints of the correctional environment. Implementing a longer or repeated program phase would also have required substantial logistical and financial resources, as participants had to be released from their regular work or educational duties during the week. Given these contextual conditions, methodological choices prioritized ecological validity and practical feasibility [43].

## 3. Results

### 3.1. Anthropometric Characteristics

Table 2 presents the anthropometric characteristics assessed at pre- and, where applicable, at post-test. These variables were assessed for descriptive purposes only and were not included in the main outcome analyses. As expected, given the short program period and the fact that the riding program was not designed to improve body composition, pre- and post-test values were largely comparable across body weight, fat mass, and fat-free mass.

### 3.2. Riding Performance

**Figure 2:**
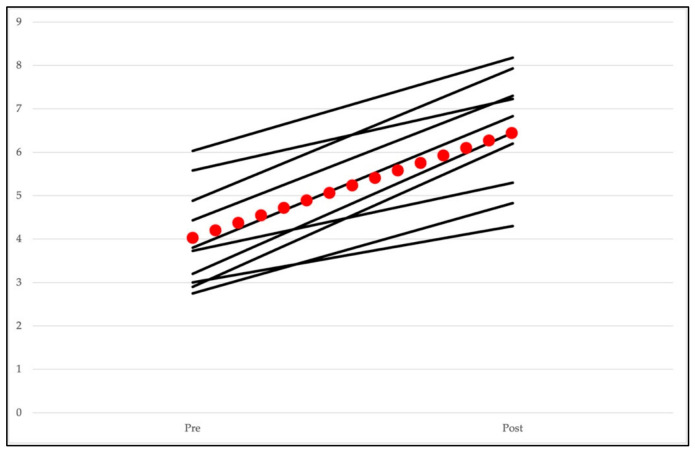
Pre- and post-test riding performance. Note. Each black line represents an individual participant. The red line with dots shows the mean value for the whole group.

As illustrated in Figure 2, the line plots depict participant trajectories, indicating a consistent overall improvement in riding performance following the program. This visualization allows for a more nuanced understanding of individual response patterns beyond mean differences. The red line with dots represents the group mean for the entire sample.

Table 3 displays the pre- and post-test values for riding performance, including the corresponding test statistics and effect sizes. The comparatively large variance in pre-test trot performance likely reflects the fact that all participants were novice riders and that the rising trot posed a substantially greater coordinative challenge than the walk at baseline. While some participants were already able to perform basic elements of the movement pattern, others showed marked initial difficulties, resulting in greater variability at pre-test.

Wilcoxon signed-rank tests indicated significant improvements in riding performance from pre- to post-test across both gaits. In the walk, riding scores increased significantly, indicating a large effect. In the trot, performance also improved significantly, likewise reflecting a large effect. When aggregated across both gaits, the overall riding performance score showed a significant increase from pre- to post-test, again indicating a large effect.

### 3.3. Movement Quality and Well-Being

Table 4 summarizes the results for movement quality, including the corresponding Wilcoxon test statistics and effect sizes. No significant change was observed for the telic dimension, whereas a significant improvement was found for the autotelic dimension, indicating a large effect.

Table 5 displays the pre- and post-test values for well-being, together with the corresponding test statistics and effect sizes. The analyses also indicated a significant increase in well-being from pre- to post-test, with a large effect.

Although several observed effect sizes were large, they should be interpreted with caution, as effect size estimates in small samples may appear comparatively large and may be less stable.

## 4. Discussion

The aim of this study was to examine whether participation in a one-week equine-assisted riding program was associated with changes in riding performance, movement quality, and well-being among young inmates in an open German prison. The results showed statistically significant and practically meaningful improvements in riding performance across both gaits. In addition, participants reported enhanced autotelic aspects of movement quality and higher levels of well-being after the program. These findings suggest that even a short equine-assisted program may be associated with measurable motor and psychological benefits in a correctional context.

The marked improvement in riding performance suggests that the participants were able to rapidly acquire fundamental motor and coordinative skills associated with horseback riding. Riding integrates postural control, rhythm, and dynamic balance, requiring continuous synchronization between human and animal motion. The significant gains observed within only five consecutive days point to the strong learning potential of experiential equine-assisted training. These improvements are particularly plausible given that all participants were complete novices without any prior riding experience, starting from a similar baseline level. This corresponds with previous findings showing that individuals with lower initial skill levels tend to exhibit greater relative performance gains during short-term motor learning [44]. An additional explanation should also be considered. Because all participants were novice riders, part of the observed improvement may have been related not only to motor learning in a narrower sense, but also to reduced fear, increasing familiarity with the horse, and greater situational confidence over time. In this regard, the pre–post gains may reflect both coordinative adaptation and a gradual reduction in anxiety within the riding situation [45].

Beyond measurable improvements in riding performance, participants also showed enhanced autotelic movement quality, reflecting a more positive and self-rewarding perception of their own movement execution. This pattern suggests that the inmates not only improved technically but also experienced the activity as intrinsically meaningful and enjoyable. Such experiential engagement is consistent with the theoretical model of movement quality [46]. The combination of focused physical training and emotional connection with the horse may have contributed to mindfulness and self-awareness, which have been shown to enhance self-regulation, intrinsic motivation, and self-efficacy, thereby supporting optimal functioning in physical activity and sport contexts [47]. Unlike purely technical training, working with horses provides immediate, embodied feedback that depends on mutual trust and attunement, allowing participants to experience cooperation rather than dominance [30].

Interestingly, the telic, goal-oriented dimension of movement quality did not show a comparable improvement, despite the objectively measurable gains in riding performance and the increase in autotelic, experience-oriented quality. This discrepancy may reflect a limited self-awareness of technical progress among participants. While the external expert ratings indicated clear motor learning, the inmates themselves may not have perceived these improvements as mastery or skill acquisition. From an educational perspective, this may also relate to the way beginners define success in their initial riding experiences. For novice riders, simply staying in the saddle and maintaining balance rather than demonstrating refined technique often represents a primary indicator of success. Moreover, beginners typically lack explicit quality criteria for evaluating their movement execution, and the horse’s perspective or feedback may initially hold little relevance for them [29]. Additionally, incarcerated individuals often experience reduced self-efficacy and a general distrust of their own abilities due to the loss of autonomy and external control typical of prison life [48]. Consequently, even when performance objectively improves, this advancement may not yet translate into an internalized sense of competence. The pattern observed in this study corresponds to theoretical assumptions on movement quality, according to which the autotelic dimension reflects the experiential quality of movement execution, while the telic dimension is more closely linked to conscious performance evaluation [49,50]. The significant improvement in autotelic quality aligns with the objectively better expert ratings, whereas the telic dimension did not increase, possibly because participants had not yet internalized their technical progress as an explicit performance gain. These findings are also in line with previous research showing that autotelic aspects correlate with the qualitative execution of movement, while telic aspects are more strongly related to measurable performance parameters such as distance or speed [34].

Well-being also improved significantly from pre- to post-test, indicating that equine-assisted activities may contribute to emotional stabilization and positive affect even within the restrictive environment of a correctional institution. These findings align with previous studies reporting that interaction with horses can reduce stress, promote empathy, and enhance psychological resilience [17,18]. The structured daily routine, cooperative group setting, and opportunity to experience autonomy and success likely supported these improvements. In this sense, the program may have provided a rare context for positive embodiment and emotional release within an otherwise monotonous and controlled environment.

While the program’s general potential to enhance psychosocial outcomes among young inmates has been reported previously [16,17,18], the present study adds a new dimension by providing the first quantitative evidence of short-term motor learning effects within an equine-assisted correctional context. These findings suggest that equine-assisted programs may support embodied learning processes that integrate physical, cognitive, and emotional components. The fact that significant improvements emerged after only one week may indicate the rapid adaptability and learning potential of experiential equine-assisted training. In doing so, this study helps to address the research gap identified in recent reviews [19], which emphasized the scarcity of quantitative analyses of motor outcomes in equine-assisted prison programs. The contribution of this study therefore lies not in confirming that equine-assisted programs can be beneficial, but in showing that such motor and experiential changes were observable within a short period, even under the constrained conditions of a correctional environment.

From a practical perspective, the findings suggest that short equine-assisted riding programs may represent a useful complementary approach in rehabilitation-oriented correctional settings. Such programs may create structured opportunities for young inmates to engage in coordinated movement, experience competence and enjoyment, and participate in cooperative, responsibility-based activities. This may be especially valuable in institutional contexts where access to meaningful physical, emotional, and social learning experiences is often limited.

The specific context of the correctional setting created several limitations for this study. The main limitation of this study was the small sample size, owing to the limited number of eligible and interested participants in the prison. Although the prison has a total capacity of 50 inmates [48], only 19 places are designated for “Jungtäter”, the target group of the present study. Of these eligible inmates, ten volunteered to participate in the riding program. The small sample size limited the statistical sensitivity of the study, and smaller effects may therefore have remained undetected. For ethical reasons, participation had to remain strictly voluntary, and no additional inmates could be required to join. As described in Section 2, this limitation was addressed by emphasizing reliable measurement procedures and transparent reporting to ensure robust findings despite restricted access to participants.

In addition, the study did not include a control group. Consequently, alternative explanations for the observed changes, such as learning effects, novelty effects, placebo-related influences, maturation, or external events within the correctional setting, cannot be ruled out. Because the program also included farm-related tasks and took place outside the prison, the broader contribution of manual work, the farm environment, and the temporary change cannot be fully separated from the observed changes, particularly in relation to well-being. Another limitation concerns the comparatively low internal consistency of the autotelic movement-quality subscale (α = 0.61). This indicates limited reliability, and the corresponding findings should therefore be interpreted with caution.

The study’s short duration and the absence of follow-up assessments limit conclusions about the sustainability of the observed effects. Because the program lasted only one week and the post-test was conducted immediately after the program, it remains unclear whether the improvements in riding performance, movement quality, and well-being were maintained or further developed over time. As outlined in Section 2, extending the program or including follow-up measurements was not feasible due to logistical, financial, and ethical constraints within the correctional environment. Future research should therefore examine the long-term consolidation of motor and psychological outcomes through extended or repeated programs and delayed post-assessments [51]. Future studies with larger samples should also examine more directly whether changes in riding performance are associated with changes in well-being and other psychosocial outcomes. In line with the chosen field-based approach, the study did not include detailed biomechanical or physiological measurements. While this limits the precision of the physical data, it ensured high ecological validity and feasibility within the correctional context [52].

Finally, the study was conducted in a single open German prison and included only young male inmates. Therefore, the generalizability of the findings is limited. Future research should include female participants, different age groups, and additional correctional facilities, including closed institutions, to assess whether similar effects can be observed across various correctional contexts.

## 5. Conclusions

The findings suggest that a short equine-assisted riding program is associated with improvements in riding performance, positive movement experience, and well-being among young inmates. From a practical perspective, such programs may represent a promising complementary approach in correctional settings by combining physical activity with emotional, social, and experiential learning opportunities.

## Figures and Tables

**Table 1:** Items of movement quality.

Item (Scale 1–6)	M	SD	Cronbach’s α
Autotelic subindex	5.13	0.66	0.610
unpleasant—pleasant	5.40	1.07	
ugly—beautiful	5.90	0.32	
hesitant—fluid	4.50	1.16	
tense—relaxed	4.70	1.06	
unstable—balanced ^1^	4.20	1.03	
Telic subindex	4.98	0.77	0.765
unsuccessful—successful	5.80	0.63	
wrong—right	4.90	1.10	
faulty—perfect	4.70	1.16	
failed—accomplished	5.20	0.79	
unrhythmic—rhythmic	4.30	1.49	

^1^ Item unstable—balanced was theoretically assigned to the autotelic dimension but showed a low corrected item-total correlation (r = 0.20) and slightly improved internal consistency when excluded (α = 0.610 vs. 0.588). Therefore, it was omitted from the reliability and index calculations.

**Table 2:** Anthropometric characteristics before and after the program.

Parameter	Pre-Test	95% CI	Post-Test	95% CI
Height (cm)	177.10 ± 5.63	[173.08, 181.12]	-	-
Body mass (kg)	87.11 ± 10.00	[79.95, 94.27]	87.75 ± 10.12	[80.51, 95.00]
Fat mass (kg)	19.89 ± 8.24	[14.00, 25.78]	19.97 ± 8.38	[13.98, 25.96]
Fat-free mass (kg)	67.22 ± 6.64	[62.47, 71.97]	67.78 ± 6.37	[63.23, 72.33]

Note. Values are presented as *M* ± *SD*; *CI* = confidence interval; *n* = 10.

**Table 3:** Riding performance before and after the program.

Parameter	Pre-Test	95% CI	Post-Test	95% CI	*z*	*p*	*r*
Walk	5.58 ± 0.61	[5.14, 6.02]	6.78 ± 0.65	[6.32, 7.24]	2.810	0.005	0.890
Trot	2.48 ± 1.92	[1.10, 3.85]	6.13 ± 2.08	[4.64, 7.61]	2.805	0.005	0.887
Overall	4.03 ± 1.16	[3.20, 4.86]	6.45 ± 1.30	[5.52, 7.34]	2.803	0.005	0.886

Note. Values are presented as *M* ± *SD*; *CI* = confidence interval; *z* = standardized test statistic; *r* = effect size; *n* = 10.

**Table 4:** Movement quality before and after the program.

Parameter	Pre-Test	95% CI	Post-Test	95% CI	*z*	*p*	*r*
Autotelic	5.13 ± 0.66	[4.65, 5.60]	5.63 ± 0.24	[5.45, 5.80]	2.552	0.011	0.807
Telic	4.98 ± 0.77	[4.43, 5.53]	5.14 ± 0.48	[4.80, 5.48]	0.653	0.514	0.206

Note. Values are presented as *M* ± *SD*; *CI* = confidence interval; *z* = standardized test statistic; *r* = effect size; *n* = 10.

**Table 5:** Well-being before and after the program.

Parameter	Pre-Test	95% CI	Post-Test	95% CI	*z*	*p*	*r*
Well-being	13.40 ± 6.70	[8.60, 18.20]	18.40 ± 7.26	[13.21, 23.59]	2.201	0.028	0.696

Note. Values are presented as *M* ± *SD*; *CI* = confidence interval; *z* = standardized test statistic; *r* = effect size; *n* = 10.

## Data Availability

The data presented in this study are available on request from the corresponding author due to the vulnerability of the participant group.

## Abbreviations

The following abbreviations are used in this manuscript:

WHO	World Health Organization
